# Supplementary material for: The Risk of Avascular Necrosis Following the Stabilization of Femoral Neck Fractures: A Systematic Review and Meta-Analysis
Source: Int J Environ Res Public Health. 2022 Aug 15;19(16):10050. doi: 10.3390/ijerph191610050 (PMC9408780; doi:10.3390/ijerph191610050)
Supplement: Supplementary file 1 [file ijerph-19-10050-s001.zip › ijerph-1756745-supplementary.pdf]

PRISMA 2020 for Abstracts Checklist

Section and Topic	Item #	Checklist item	Reported (Yes/No)
**TITLE**			
Title	1	The risk of avascular necrosis following stabilisation of femoral neck fractures: a systematic review and meta-analysis.	Yes
**BACKGROUND**			
Objectives	2	We conducted a systematic review of recent research into the risk of avascular necrosis (AVN) following stabilisation of a fractured femoral neck with implants.	Yes
**METHODS**			
Eligibility criteria	3	Inclusion criteria:<br>- original research articles describing real-world studies reporting on the risk of AVN following primary surgical fracture stabilisation with implants<br>- published in English<br>- published between 1 January 2011 and 22 April 2021<br>Exclusion criteria:<br>- case reports<br>- case series of less than 11 cases<br>- studies focusing on patients with neglected fractures	Partly (due to limited space)
Information sources	4	PubMed Database, last searched on 11 May 2021	Partly (due to limited space)
Risk of bias	5		No
Synthesis of results	6	The meta-analysis results are presented as pooled means with 95% confidence intervals (CIs)	No
**RESULTS**			
Included studies	7	A total number of 52 studies (N=5930) were included, most of them were retrospective studies	Partly (due to limited space)
Synthesis of results	8	The pooled mean AVN incidence was significantly higher among patients with displaced fractures (20.7%; 95% CI: 12.8%-28.5%) than among those with undisplaced fractures (4.7%; 95% CI: 3.4%-6.0%) but did not significantly differ between the two age groups. We found no significant differences in AVN risk by the fracture Garden stage or Pauwels degree, but the analysis of the latter was based on few studies. We observed a non-significant trend for increasing AVN risk with higher Delbet stages. There was no significant correlation between AVN incidence weighted by sample size and the time elapsed from injury to surgery.	Partly (due to limited space)

PRISMA 2020 for Abstracts Checklist

Section and Topic	Item #	Checklist item	Reported (Yes/No)
**DISCUSSION**			
Limitations of evidence	9	The main limitations of this review is a small number of studies with data on fracture types (Pauwels and Delbet classifications)	No
Interpretation	10	The risk of AVN following femoral neck fractures was generally high and it was significantly higher for patients with displaced fractures than for those with undisplaced fractures. Therefore, a long-term post-surgical follow-up of these patients is necessary. The time from injury to surgery did not correlate with AVN incidence.	Partly (due to limited space)
**OTHER**			
Funding	11		
Registration	12	The review has not been registered in any database.	No

From: Page MJ, McKenzie JE, Bossuyt PM, Boutron I, Hoffmann TC, Mulrow CD, et al. The PRISMA 2020 statement: an updated guideline for reporting systematic reviews. BMJ 2021;372:n71. doi: 10.1136/bmj.n71

For more information, visit: http://www.prisma-statement.org/
